# Naming, Stimulus Equivalence and Relational Frame Theory: Stronger Together than Apart

**DOI:** 10.1007/s40614-024-00427-z

**Published:** 2025-01-09

**Authors:** Alceu Regaço, Colin Harte, Dermot Barnes-Holmes, Julian Leslie, Julio C. de Rose

**Affiliations:** 1 Ulster University, Coleraine, Northern Ireland UK; 2 Universidade Federal de São Carlos, São Carlos, Brazil; 3 Instituto Par, São Paulo, Brazil; 4 Instituto Nacional de Ciência e Tecnologia Sobre Comportamento, Cognição e Ensino, São Carlos, Brazil

**Keywords:** Derived relations, Stimulus equivalence, Relational frame theory, Naming theory, Verbal behavior

## Abstract

Research on human language started to change when Murray Sidman and colleagues demonstrated that a participant was able to derive unreinforced stimulus relations after conditional discrimination training. This work provided the basis for a novel approach to research on symbolic behavior and fostered the development of three main theoretical accounts: stimulus equivalence (SE), relational frame theory (RFT), and naming theory (NT). These accounts unfolded in the last decades of the twentieth century, promoting intense debate and discussion within behavior analysis. Although experimental research emerging from these three accounts is still highly active today, the theoretical discussions have, to a large extent, faded. Considering the importance of rekindling a dialogue, this article aims to describe the differences among the three accounts, but focus on their common points. We conclude by arguing that developing a more complete behavior-analytic account of human language would be served best by considering both research and theoretical analyses of SE, RFT and NT. Finally, we provide examples of two successful research groups that adopted this approach and in doing so have advanced our understanding of language within behavior analysis.

Language is one of the most complex research fields within behavior analysis. Skinner’s approach, referring to it as verbal behavior (VB), “emphasizes the individual speaker” (Skinner, 1957, p. 2), by focusing on the variables that control the emission of verbal responses. He defined VB based on the mediation of consequences provided by listeners who had been conditioned to respond in that way. Furthermore, Skinner argued that the same behavioral principles from research with nonhuman behavior would be largely sufficient to explain and describe human VB (Michael, 1984). Skinner also argued that listener behavior “has no resemblance to the behavior of the speaker and is not verbal according to our definition” (Skinner, 1957, p. 33), and later introduced the concept of rule-following behavior to describe how language, or verbal stimuli, can control behavior (Skinner, 1966, 1969). He described rules as contingency-specifying stimuli, the effects of which could be explained by the contingencies that shaped both rule-following behavior in general and the particular behavior specified in a given rule. This definition resulted in numerous empirical studies and conceptual analyses, and even a full book-length treatment (Hayes, 1989). Much of this work focused on the differences between rules and direct contingency-shaped behavior, and variables that increase or diminish rule-following behavior (e.g., de Almeida et al., 2020; Galizio, 1979; Harte et al., 2020; Hayes et al., 1986; Zapparoli et al., 2021).

Another research line on language within behavior analysis started with an experiment conducted by Murray Sidman, which was designed to teach reading with comprehension to a neurodivergent teenage male (Sidman, 1971). At first, the participant was only able to point to the images when the names of the images were presented to him (e.g., pointing to a picture of a cat when hearing the word “cat”). But after conditional discrimination training to establish a relation between the spoken name and the written word (e.g., pointing to the word cat upon hearing “cat”), the teenager derived other relations without the need of additional training (e.g., he could point to the image when seeing the word, point to the word when seeing the image, and say the name when presented with the word). Sidman subsequently developed an account based on mathematical set theory to describe the emergence of the derived relations, in what was later termed the stimulus equivalence (SE) paradigm (Sidman, 1994; Sidman & Tailby, 1982).^1^ These authors stated that a class of equivalent stimuli would be documented when, after a baseline training (e.g., A → B and B → C), a subject could respond in terms of reflexivity (e.g., A → A, B → B, and C → C), symmetry (e.g., B → A and C → B), and transitivity (e.g., A → C) in the absence of direct reinforcement. That is, for SE, classes of equivalent stimuli are defined based on the derivation of multiple stimulus relations that attest the defining properties of equivalence relations according to the mathematical definition (i.e., reflexivity, symmetry, and transitivity).

Sidman and his colleagues argued that the phenomenon of stimulus equivalence may provide a functional-analytic definition of symbolic meaning or reference. That is, a functional definition of a symbol requires that it participate in an equivalence class. In the decades that followed, the equivalence paradigm as a conceptual analysis of symbolic meaning, combined with the economy of teaching generated by the emergence of multiple untrained relations, led to the development of many lines of research (e.g., Arntzen, 2012; Brodsky & Fienup, 2018; de Rose et al., 1996; Fields, 2023; Regaço et al., 2023; Sidman, 1994).

Two other conceptual accounts of human language emerged from Skinner’s VB, and from Sidman’s SE: relational frame theory (RFT) and naming theory (NT). RFT emerged initially out of an attempt to explain rule-governed behavior, drawing on the concept of stimulus equivalence and other patterns of derived stimulus relations (e.g., different, same, comparison), and aiming to develop a general behavior-analytic account of human language and cognition (Hayes, 1989; Hayes et al., 2001a, 2001b). The authors argued that language, or complex VB, is rooted in a type of generalized operant described as arbitrarily applicable relational responding (AARR). The core idea is that AARR differs from other types of relational responding because it is not controlled solely by the nonarbitrary or formal characteristics of the related stimuli (Hayes, 1991a, 1991b). As an example, in Sidman’s (1971) first equivalence study, there were no formal characteristics of the spoken word “cat” that related it to the written word *cat*. Hayes et al., (2001a, 2001b) also suggested that equivalence, or coordination, is one of several patterns of generalized relational responding (e.g., opposition, temporal, and deictic) that could be brought under contextual control. In addition, RFT emphasized the role of multiple exemplar training (MET) on the development of AARR, and the role of contextual cues in determining and controlling derived relational responding (Hayes et al., 2001a, 2001b). Therefore, in opposition to Skinner’s view of listener behavior as nonverbal, and aligned with Sidman’s SE research, RFT proposed that both speaker and listener behavior are verbal, and that AARR is the core functional-analytic basis of human language and cognition, including problem-solving (Hayes, Gifford et al., 2001) and instructional control (Barnes-Holmes et al., 2001a, 2001b; Hayes et al., 2001a, 2001b).

Another line of inquiry that emerged from SE was NT, first proposed by Horne and Lowe (1996). In particular, these authors aimed to show how some of the concepts proposed in Skinner’s *Verbal Behavior* (1957) could be employed in constructing an account of the emergence of naming and its role in symbolic meaning, stimulus equivalence, and verbal categorization in general (e.g., Horne et al., 2004; Lowe et al., 2002, 2005). The authors described naming as a higher order behavioral relation that combines speaker and listener behavior within the same individual, resulting from the combination of listener, echoic, and tact responses.^2^ According to this account, after naming is established, reinforcing a listener response results in the emergence of a speaker response, and reinforcing a speaker response results in the emergence of a listener response, without the need for direct reinforcement of both responses. Horne and Lowe (1996) stressed the fundamental role of listener behavior for the development of linguistic behavior and focused on the importance of bidirectionality between classes of objects and speaker/listener behaviors. In addition, the authors argued that naming is necessary for the demonstration of equivalence responding. In a typical matching-to-sample (MTS) procedure, the participant tacts the sample stimulus, the stimulus name is then echoed until the presentation of the comparison stimuli, ultimately controlling the listener response of pointing to the correct comparison stimulus. Horne and Lowe (1996) also indicate how, in their view, intraverbal relations may be a part of this process when different names are given for stimuli in the same equivalence class (Miguel, 2016). However, it is important to note that Lowe and Horne (1996) acknowledged that equivalence may occur based on unspecified nonverbal contingences (p. 329).

## Debate among SE, NT, and RFT

From the late 1980s to the early 2000s, there was considerable debate among researchers concerning these three different approaches (SE, RFT, NT) to derived stimulus relations and human language and cognition more generally (e.g., Clayton & Hayes, 1999; Hayes & Barnes, 1997; Lipkens et al., 1993; Sidman, 2000; Sidman et al., 1986; see also responses to Horne & Lowe, 1996, and their reply). Although the intensity of the original debate has more or less died away, the three broad lines of research remain highly active but separate today (e.g., Barnes-Holmes & Harte, 2022; Fienup & Brodsky, 2020; Greer et al., 2024; Hayes & Hofmann, 2023; Miguel, 2018; Petursdottir, 2023; Regaço et al., 2023; Sivaraman & Barnes-Holmes, 2023). On balance, it is important to note that there appears to be an ongoing debate among researchers that use VB concepts to explain stimulus equivalence but do not fully agree with the NT description of naming as a generalized/higher-order operant (e.g., joint control; Lowenkron, 1998, 2006; see also Michael, 1996). In addition, verbal behavior development theory (VBDT), which emerged from NT, and emphasizes the role of naming in language development, extends very much beyond this focus (Greer & Speckman, 2009; we will return to VBDT in particular at a later point). Nevertheless, despite these divergences, we will use the term NT as a broad category that encompasses (or exemplifies) these research traditions in remaining closely aligned with Skinner’s VB concepts as a platform from which to understand stimulus equivalence.

Although some authors have attempted to bring the various approaches to language and cognition together (Barnes-Holmes et al., 2000; Greer & Speckman, 2009; Perez, 2023; Petursdottir, 2023; Sivaraman et al., 2023), it could be argued that their respective research activities rarely interact in a collaborative or mutually supportive manner. Indeed, the situation is such that it has been proposed that the different approaches may be best seen as separate “model dependent” accounts of different aspects of human language and cognition (Belisle, 2020). The current article aims to discuss possible reasons for this lack of interaction among approaches, consider their points of agreement and disagreement, and question whether or not this separation is beneficial for behavior analysis in general.

## Reasons for the Separation among SE, NT, and RFT

### Social Factors

One possible reason for the division among these three main theories might be a degree of scientific conservatism regarding the proposal of new concepts that diverge from previously established ones (Gross & Fox, 2009), particularly before existing accounts have been fully and completely tested (although what defines “fully and completely tested” will always remain a matter of debate; e.g., Schlinger et al., 2024). Skinner wrote a full-length book as an interpretative exercise in applying basic behavioral processes, mostly wrought from nonhuman research to human language (Skinner, 1957). The importance of Skinner’s analysis and the influence his work had on behavior analysis was significant in the applied domain, especially with children with language deficits (e.g., Sundberg & Michael, 2001). However, Skinner’s book was not concerned with language acquisition, and its emphasis on a direct contingency^3^ (i.e., explicitly reinforced) account of VB was difficult to align with Sidman’s first experiments on stimulus equivalence, in which the participant did not have a complex verbal repertoire, but still derived new relations that were not directly reinforced (Sidman, 1994, p. 21). According to Sidman (1994), the derivation of new relations was an unexpected result (if one assumes that all instances of stimulus control over operant behavior require an appropriate history of direct reinforcement), and as such, the phenomenon of stimulus equivalence appeared to require a treatment that extended beyond the principles of behavior analysis available at that time.

Although researchers in both SE and RFT clearly stated that their accounts were developed within a behavior-analytic paradigm (Hayes et al., 2001a, 2001b; Sidman, 1994), it is understandable that others in the field were somewhat skeptical of this (see the exchange between Sidman and Day; Sidman, 1994, p. 561). A more conservative approach would be to attempt to explain the new findings on derived relations research using previously established concepts in the study of human language, i.e., Skinner’s VB operants. Indeed, one of the main aims of NT was to explain stimulus equivalence using Skinner’s account, with the authors arguing that “Verbal Behavior should be seen as providing a vital platform for further theoretical development” (Lowe & Horne, 1996, p. 316). However, the question of whether Skinner’s VB was adequate in accounting for the new findings of SE was a matter of both theoretical interpretation and experimental research. Furthermore, the NT interpretation was questioned by some researchers who pointed out that Skinner’s account was not designed to address concepts such as reference and symbolic meaning (de Rose, 1996; Hayes, 1991a, 1991b; Parrott, 1984), whereas SE appeared to address exactly these concepts and in a functional-analytic way (Sidman, 1994).

In this scientific context, one can appreciate the wider social contingencies at play. The most straightforward and conservative strategy to adopt in the face of Sidman’s SE and its close connection to language (i.e., reading) was to use an already established account in that domain (i.e., VB). And this is what NT attempted to do. Those researchers who questioned whether VB could provide an account of stimulus equivalence could thus be seen as intellectual “renegades” who reached too quickly for an alternative explanation. However, it should be noted that NT itself was not a straightforward application of Skinnerian concepts, but it involved constructing the new behavioral unit of naming by combining the concepts of listener, tact, and echoic. Skinner did not refer to naming as a verbal operant, and he did not suggest that verbal operants could merge into a general operant. So, although NT could be seen as conceptually more aligned with VB, it did involve the introduction of a new behavioral unit to explain stimulus equivalence (and this was criticized by other VB researchers; see Michael, 1996, as an example). Furthermore, the concept of naming as an explanation for stimulus equivalence remained an empirical issue, and thus there was scope for others to propose alternative accounts that would also, of course, require empirical evidence (Clayton & Hayes, 1999).

Overall, the social/scientific context at that time, and arguably to this day, could be seen as encouraging the proposal of competing theories to account for stimulus equivalence. And indeed, this is what happened: within the behavior-analytic community three intellectual tribes emerged, each defending their own theoretical and conceptual territory, and seeking to produce evidence to support their own account and refute others. It is perhaps regrettable that in the heat of battle, the prize of developing a coherent behavior-analytic account of stimulus equivalence, and human language in general, was perhaps lost or at least shuffled off center stage (Hayes, 1996). It was in this context of intellectual disagreement that differences in the content and focus of the accounts perhaps fed back into maintaining and exaggerating these differences, an issue to which we now turn.

### Differences in Content

Researchers aligned with each of the three theoretical approaches have made significant progress in the past 3 decades. SE researchers focused on the variables that facilitate the emergence of equivalence classes (e.g., Arntzen, 2012; Fields, 2023), with a special emphasis on the application of SE to education (e.g., Brodsky & Fienup, 2018; Fienup & Brodsky, 2020; Pilgrim, 2020). NT researchers conducted numerous studies to understand the relation between naming and equivalence (e.g., Miguel, 2018; Petursdottir, 2023; see Sivaraman & Barnes-Holmes, 2023, for a recent relevant review), to explore the role of naming on early language development (e.g., Greer & Speckman, 2009; Greer et al., 2024), and to refine NT itself (Hawkins et al., 2018; Miguel, 2016). And RFT researchers focused on the broad domain of language and cognition, including rule following (e.g., Harte et al., 2020), analogical reasoning (e.g., Stewart & Barnes-Holmes, 2004), intelligence (e.g., Cassidy et al., 2016), and perspective-taking (e.g., Edwards et al., 2017; for a broader review of research on RFT in the last decades, see Hayes et al., 2021). In recent research, some authors have focused on developing RFT itself by considering the role of human cooperation in the emergence of AARR (Hayes & Sanford, 2014) and creating a broad framework and unit of analysis for systematizing the study of AARR (see Barnes-Holmes & Harte, 2022, for recent summary of this effort).

In very broad terms, NT and VB experimental research has been focused primarily on vocal verbal behavior, most frequently studied in young children, including those with language deficits (Petursdottir, 2018; Sivaraman & Barnes-Holmes, 2023). In a systematic review on naming, Sivaraman and Barnes-Holmes (2023) identified 46 studies, 43 of which (93%) included children as participants, with 67% categorized with a developmental disability. In another review, by Petursdottir and Devine (2017) on VB experiments from 2006 to 2015, the studies included children exclusively in 84% of the cases, and 72% were clinical populations (largely focused on autism).

Also in very broad terms, experimental research on SE and RFT has tended to focus on adult/college populations (Belisle, 2020; Dymond et al., 2010; O’Connor et al., 2017), mostly using conditional discrimination procedures, such as MTS. Two systematic reviews on RFT, including the period between 1981 to 2008 (Dymond et al., 2010), and 2009 to 2016 (O’Connor et al., 2017), indicated that the population studied was primarily adults (72% and 78%, respectively). And although there is no current broad review on SE, a preliminary review of SE experiments from 2000 to 2018 showed that 53% of the participants were college students (Belisle, 2020). Nevertheless, considering the scope and the number of studies conducted on SE, it seems likely that there is more diversity in the populations studied (including nonhuman species) than in the other research traditions.

The summary provided by the systematic reviews, on balance, may not present the full picture of studies in SE, RFT, and NT. Although O’Connor et al. (2017) indicated that most research in RFT was conducted with adults, they still identified 64 published studies with children, including both typical and neurodiverse populations. And the same applies to the numerous experiments conducted both by NT and VB researchers, who studied adult and complex language phenomenon. Indeed, there have been NT studies on complex nonvocal responses (e.g., Frampton et al., 2023); and there have been RFT (e.g., Lipkens et al., 1993; Pomorska et al., 2021) and SE (e.g., Almeida-Verdu et al., 2021) studies on early language development.

In this context, therefore, although there are clearly numerous exceptions, research conducted in NT and VB has been more focused on early language development in children, and research in SE and RFT has placed a greater emphasis on more complex language phenomena, typically associated with adult studies. Nevertheless, at least theoretically, the aims of those different approaches could be seen as more broadly aligned. For example, from the earliest days of NT, Horne and Lowe (1996) argued: “Although we have concentrated upon how naming, the most basic verbal unit, comes about during the first 2 years of life, *our approach and method could be extended to account for the full range of linguistic behavior*” (p. 240; emphasis added). Furthermore, early language development, and naming in particular, were always considered central to the RFT explanation of how equivalence (or frames of coordination) emerges in the behavior of young children. Indeed, naming was considered one of three pillars of human language and cognition within the theory (Hayes et al., 2001a, 2001b). And although some equivalence researchers questioned the explanatory role of naming in equivalence responding, they certainly did not claim that it was irrelevant (Sidman, 1994).

Overall, therefore, it appears that all three accounts developed with the focus on understanding human language in whole cloth, and it may be unwise to perpetuate the view that specific approaches are *only* concerned with early verbal development, or alternatively *only* with complex “adult” language phenomena. In any case, the general initial focus of each approach on different populations and procedures very likely contributed to the growing separation among the researchers involved in each approach, despite the clear areas of overlap in their research interests. And it is regrettable that it is perhaps in this context that theoretical differences were amplified.

### Theoretical Differences

In the context of derived equivalence relations, there are at least two common points among SE, RFT, and NT (Clayton & Hayes, 1999; Horne & Lowe, 1996). First, that participants treat different stimuli as if they were interchangeable, depending on the context, and that it is important to establish how that happens; and second, that this phenomenon is closely related to language. In addition, it can be argued that RFT and NT share a common hypothesis of the development of a generalized operant through a learning history, whereas SE maintains that the origin of equivalence responding is the contingency itself (Sidman, 2000). Furthermore, with recent developments in RFT and NT, broadly defined, Sivaraman et al. (2023) suggested that both accounts place significant importance on the integration of speaker and listener responses, the subsequent emergence of spontaneous or unreinforced naming responses (referred to as incidental bidirectional naming), and the importance of orienting behavior in early language development. Indeed, some common points are generally acknowledged by researchers from NT, RFT, and SE (e.g., Greer et al., 2024; Saunders & Spradlin, 1996; Sivaraman et al., 2023). However, the three accounts still diverge in terms of the relation between language and stimulus equivalence. As described by Petursdottir (2023), when referring to NT and RFT: “The disagreement is, indeed, one of theoretical significance because the two perspectives imply rather different conceptualizations of language and its relationship with DRR [derived relational responding]” (p. 3). In this regard, therefore, the foundation stone of the division between the three accounts appears to be the relationship between stimulus equivalence and language.

In particular, NT researchers may have seen little relevance in SE and RFT research, given that mediational behavior (generally vocal) was not explicitly considered in the interpretation of research findings. As an example, Sundberg et al. (2018)^4^ argued that “proponents of equivalence theory and RFT tend to dismiss the relevance of a participant’s mediating verbal behavior as a source of stimulus control when examining emergent relational behavior” (p. 617). On balance, although SE researchers seemed less interested in vocal/mediational accounts, they did not dismiss the potential importance of mediation per se. Thus, Sidman (1994) argued that “to say that verbal mediation is unnecessary for equivalence is not to say that verbal labels and rules are always irrelevant” (p. 511). Furthermore, RFT researchers recognized that verbal mediation may well occur, but that this was behavior to be explained rather than an explanation in and of itself (Hayes & Barnes, 1997). As argued by the authors:

We do not deny, of course, that subjects may name the relations involved, but in terms of relational frame theory this shows only that stimulus relations themselves can enter into a “frame of coordination” with a name. It does not show why named relations relate. (Hayes & Barnes, 1997, p. 240)

In short, NT researchers sought to interpret research on SE and RFT as involving complex combinations or interactions among Skinner’s VB concepts. From this perspective, therefore, language phenomena would be better understood as an interaction of verbal operants in increasingly complex ways (see Sundberg et al., 2018, for a description of the operants needed and their interaction) or as a type of problem-solving behavior (Miguel, 2018). In contrast, SE researchers argued that they were studying the symbolic properties of language (Sidman, 1994), which could be assessed independently of vocal mediation, while also affirming that verbal mediation may play a role in SE, although not necessarily a causal one (e.g., Saunders & Spradlin, 1996). Finally, RFT researchers argued that *both* naming and stimulus equivalence were examples of the early development of AARR, and as such were inherently part of verbal behavior (e.g., Hayes & Barnes, 1997; Hayes et al., 2001a, 2001b).

As described above, Petursdottir (2023) argued that the main point of division between the three accounts is the relation between language and stimulus equivalence. Sidman (1994) also came to that conclusion in one of his letters to Willard Day, when discussing the implications of SE:

An important question that I have not yet resolved is the explanatory status of equivalence relations. It is a "which comes first, chicken or egg?" kind of problem. Do equivalence relations help us to explain some aspects of verbal behavior—for instance, the "specification" of contingencies by rules—or does verbal behavior—for example, rules—make equivalence relations possible? Most people believe the latter (you, too, I would guess from the first part of your ABA paper), but I am not certain; surely, verbal chains can mediate equivalence relations, but are they necessary? (p. 567)

After more than 30 years and many experimental investigations, it is still not possible to answer definitively that question, although it does appear that language (or verbal behavior) is closely related to stimulus equivalence (or derived relational responding). It is interesting to note that a number of authors have questioned whether it will ever be possible to prove or disprove NT in favor or against SE and/or RFT, based on experimental inquiry alone (e.g., Dickins & Bentall, 1996; Fields, 1996; Pilgrim, 1996; Saunders & Spradlin, 1996).

In this context, therefore, it is reasonable to question whether further research aimed *only* at proving or disproving one account over the other will lead to any major change in the general understanding of language within behavior analysis, particularly when considering the numerous common points among the three accounts. As one example, experiments designed *solely* to gather evidence that stimulus equivalence (or other types of derived relational responding) may occur when verbal responses are taught/required may not provide substantial conceptual advances. That is, SE and RFT do not disagree that stimulus relations may emerge or be influenced by spoken stimuli or instructions. Indeed, derived relations may be trained and/or tested using different procedures, such as the MTS, IRAP (implicit relational assessment procedure; e.g., Bortoloti & de Rose, 2012; Murphy et al., 2019), respondent type procedures (e.g., Ribeiro et al., 2020), go/no-go (e.g., Debert et al., 2007), and sorting (e.g., Arntzen et al., 2015), with each providing important subtleties in focus, but none fully encompassing the phenomenon of interest (Clayton & Hayes, 2004; Marin & Fienup, 2024).

Indeed, pursuing a “which theory is best” approach, at the expense of focusing on the common points, may lead to even more separation and to research designed solely to prove one account as “more correct” or “more parsimonious” than the others (e.g., Alonso‐Álvarez & Pérez‐González, 2017, 2021; Schlinger & Blakely, 2024). Consistent with this direction, Sivaraman and Barnes-Holmes (2023) pointed out that almost one third of NT studies were not focused on naming per se, but on providing evidence to show that naming was an explanation for stimulus equivalence or categorization (i.e., this research was concerned with gathering evidence to prove that NT explained stimulus equivalence over other accounts). Of course, criticism and debate among the accounts may be beneficial and should not be prohibited or discouraged, but a productive discussion should not overlook or diminish what the accounts have in common, much less minimize the progress achieved by the various experimental analyses produced by any of the accounts.

## An Alternative to Separation: Some Examples

In the general context described above, one might wonder if it will be possible to study language within behavior analysis without being dragged into the lions’ den of “which account is the best.” However, there are examples of research projects that left the dispute aside to focus on understanding language by considering what the different accounts had to contribute. Two of these cases will be further described: VBDT, and the program “Learning to Read and Write in Small Steps” (*Aprendendo a Ler e Escrever em Pequenos Passos*; ALEPP).

Greer and Speckman (2009) described VBDT as an attempt to explain the early development of verbal capabilities based on the assumption, shared by both RFT and NT, that to be truly verbal, speaker and listener repertoires should be integrated. The authors also based their work on Skinner’s VB operants and on the concept of behavioral cusps (Rosales‐Ruiz & Baer, 1997). Overall, VBDT researchers have developed a broad understanding of language development based on the establishment of different cusps, such as preverbal, listener, speaker and the joining of speaker and listener, while also defining procedures and protocols to access these repertoires (e.g., Conceição et al., 2022; Greer & Ross, 2008; Sivaraman et al., 2023). Greer and Speckman (2009) argued that to properly understand verbal development, and help children with language delay, behavior analytic research *in general* should be considered (i.e., independent of specific accounts):

One of the reviewers asked us what our position was on the different theories pertaining to emergent verbal behavior. Rather than differences, we saw amazing consistency for our purposes—the induction of verbal capabilities in children who were missing them. . . . Though there are differences in interpretation about these various phenomena, we are interested in findings and theories that work in inducing verbal behavior. (p. 478)

This position appears to have been maintained to this day, with VBDT researchers frequently pointing to the similarities and commonalities across VBDT, RFT, NT, and SE (e.g., Greer et al., 2024; Hranchuk & Greer, 2024; Sivaraman et al., 2023).

Another example of the interaction between different accounts is the ALEPP program, whose first version was presented in a meeting that reunited researchers from different accounts, including Murray Sidman and Steven Hayes.^5^ The project aimed to develop a behavior analytic solution for helping children with poor reading instruction, based in SE and VB accounts, and on Keller’s Personalized System of Instruction (PSI; Keller, 1968). In particular, some of the behavior-analytic literature that helped the formulation of ALEPP were Skinner’s theoretical analysis of “minimal units” of verbal behavior (Skinner, 1957), Mackay and Sidman’s (1984) constructed-response MTS, and SE learning by exclusion (McIlvane & Stoddard, 1981). The program went through several modifications through the years (Golfeto & Postalli, 2021), but it is currently composed of three computerized modules that teach word and sentence reading, progressing from easier words, having only syllables formed by consonant followed by a vowel (e.g., ball, *bola* in Portuguese) to words with more complex syllables (e.g., snake, *cobra* in Portuguese) and full sentences (see Albuquerque & Melo, 2021, especially Section II, for the development of the modules of the program). The ALEPP program has been applied to more than 3,000 neuro-typical Brazilian children (Pilgrim, 2020), while also being used with other populations, such as neuro-atypical (e.g., Benitez & Domeniconi, 2016, 2023; Melchiori et al., 2000), and hearing-impaired children (e.g., Lucchesi et al., 2022).

Both VBDT and ALEPP were developed with a specific research focus, but with a broad consideration of behavior analytic research on language. They were successful not only in answering their initial questions, but on fostering basic experimental research and improving behavior-analytic application to language development. And although it is not possible to define just one reason for the reach of those different projects, they stand out as examples of how a comprehensive appreciation of different accounts and experimental research within behavior analysis can be fruitful.

The general benefits accruing from the foregoing large research programs may also emerge in different areas. Indeed, studies in any one of the three theoretical areas (SE, NT, and RFT) may benefit from considering critical variables highlighted in one or both of the other areas. For example, SE research has a history of testing the effects of the simultaneous versus delayed presentation of sample stimuli in MTS procedures (Arntzen, 2012). However, the effects of delays between name and object stimuli are rarely considered within NT research. One exception is the work of Sivaraman et al. (2021), in which they tested the effects of a nonsimultaneous presentation of a stimulus and its name in a typical naming task (i.e., saying the object name and presenting the object, or presenting the object and saying its name). In their discussion, the authors pointed to the importance of determining the impact of this variable in naming research (see also Sivaraman & Barnes-Holmes, 2023).

Another example of where the different theoretical perspectives may usefully draw on each other is the impact of contextual cues on incidental naming. According to RFT, contextual cues help to establish and control relational responding between names and objects (Gilmore et al., 2024; Hayes et al., 2001a, 2001b). Indeed, Gilmore et al. (2024) argued that we may better understand how children learn names incidentally (in the absence of reinforcement) by studying different linguistic (e.g., “What is this?,” “This is a...,” “Look...”) and paralinguistic (e.g., eye gaze, pointing, facial expressions) contextual cues. Although such cues are often involved in naming research, they have never been systematically studied, thus going forward there is a potential opportunity for an integrative research line that considers both NT and RFT.

The VBDT, the ALEPP, and the examples of possible interactions among the accounts are but a few of the research possibilities when NT, SE, and RFT are taken together into consideration. Of course, it remains uncertain whether this more integrative and collaborative approach would definitely advance the field in a very substantive way, but it does seem like a healthy alternative to the current separation among the research groups.

## Conclusion

The aim of the current article was to discuss possible reasons for the relative absence of interactions among SE, NT, and RFT researchers, considering points of connection among these accounts and questioning whether this division is beneficial for behavior analysis. In doing so, we described the development of the three accounts on derived stimulus relations and suggested reasons for the differences between them in terms of social, content, and theoretical factors. Overall, the three accounts have a similar history, in that they may each be interpreted as extensions to Skinner’s VB operant account, although with different levels of agreement and disagreement with that seminal account. With this similar focus, researchers from SE, RFT, and NT focused on different populations and on different research areas, developing numerous research programs in both basic and applied settings, although largely separated from each other. When reflecting upon the theoretical differences among the accounts, however, there is perhaps one major difference: the origin of derived relational responding. In brief, SE researchers argue that stimulus equivalence is the product of the contingency itself; RFT researchers argue that it is the product of the development of a generalized operant through multiple exemplar training; and NT researchers argue that verbal mediation is necessary for derived relations to develop.

Although these differences are important and should be addressed in experimental and theoretical research, we argue here that they should not overshadow or dominate the similarities in the accounts we have outlined herein. In addition, we maintain that considering research across all accounts might be the most effective way to develop a comprehensive account of an increasing range of language phenomena. As examples of good practice, we briefly considered programs of two research groups that adopted that approach and as a result, it appears, made important progress towards the behavior-analytic understanding of language development. Finally, we hope that this article may help to elucidate the contexts in which NT, SE, and RFT were developed and we hope will foster a more comprehensible and equitable view of the different accounts on derived relations research within behavior analysis.

## Data Availability

Not applicable.

## Footnotes

1 The abbreviation SE will be used when referring to Sidman’s descriptive account of stimulus equivalence: “My own theorizing has been directed not so much at an explanation of equivalence relations but rather, at the formulation of a descriptive system” (Sidman, 1994; p. 536). And “stimulus equivalence” will be used when referring specifically to the phenomenon of two stimuli being equivalent to each other depending on the context.

2 Echoic behavior is a verbal operant that sounds like the controlling verbal stimulus (i.e., the speaker repeats a previous verbal behavior, emitted by themselves or by someone else). A tact is a “verbal operant in which a response of a given form is evoked or at least strengthened by a particular object or event or property of an object or event” (Skinner, 1957, pp. 81–82). The term “listener behavior” usually designates a nonverbal behavior that is an appropriate response to a previous verbal behavior, as for instance when the listener points to the picture of a cat (among other pictures) after hearing the word “cat” (for a detailed description see, Skinner, 1957).

3 Of course, we recognize that Skinner was aware of the possibility of derivation. As an example, he used the term “magical mand” to describe situations in which the mand “cannot be accounted for by showing that they have ever had the effect specified or any similar effect upon similar occasions” (Skinner, 1957, p. 48; see Murphy & Barnes-Holmes, 2009, for an experimental study on magical mands). He also used the term “relational autoclitic behavior” and, in particular, “autoclitic frame” to describe the derivation of verbal responses (see Skinner, 1957, p. 336). However, Skinner did not attempt to provide an account of such derived responding, probably due to the historical context in which the book was written and the lack of research on that topic at that time.

4 Although the study by Sundberg et al (2018) is not presented as a NT experiment, the authors describe NT and joint control as accounts that are to be contrasted with SE and RFT, based on the role of mediation on stimulus equivalence, providing arguments in favor of the former.

5 Procedures of this meeting, the Third International Institute on Verbal Relations (held in 1989 at the town of Aguas de Lindoia, Brazil) are provided in Hayes and Hayes (1992). They include a chapter presenting a preliminary version of what became the initial module of the APEPP program (de Rose et al., 1992). The impact of the meeting for the development of the program is recounted by de Souza et al. (2009).

## References

[CR1] Albuquerque, A. R. de, & Melo, R. M. de. (2021). *Contribuições da análise do comportamento para a compreensão da leitura e escrita: aspectos históricos, conceituais e procedimentos de ensino - Volume I*. Faculdade de Filosofia e Ciências. 10.36311/2021.978-65-5954-075-4

[CR2] Almeida-Verdu, A. C. M., das Neves, A. J., de Souza, D. G., & Postalli, L. M. M. (2021). Subsídios necessários para ampliar o programa de ensino de repertórios verbais visando sentenças. In *Contribuições da análise do comportamento para a compreensão da leitura e escrita: aspectos históricos, conceituais e procedimentos de ensino—Volume I* (pp. 249–286). Faculdade de Filosofia e Ciências. 10.36311/2021.978-65-5954-075-4.p249-286

[CR3] Alonso-Álvarez, B., & Pérez-González, L. A. (2017). Contextual control over equivalence and nonequivalence explains apparent arbitrary applicable relational responding in accordance with sameness and opposition. *Learning & Behavior,**45*(3), 228–242. 10.3758/s13420-017-0258-1 28275954 10.3758/s13420-017-0258-1

[CR4] Alonso-Álvarez, B., & Pérez-González, L. A. (2021). Equivalence class analysis of responding consistent with the relational frame of opposition. *Journal of the Experimental Analysis of Behavior,**116*(1), 64–81. 10.1002/jeab.690 33914345 10.1002/jeab.690

[CR5] Arntzen, E. (2012). Training and testing parameters in formation of stimulus equivalence: Methodological issues. *European Journal of Behavior Analysis,**13*(1), 123–135. 10.1080/15021149.2012.11434412

[CR6] Arntzen, E., Norbom, A., & Fields, L. (2015). Sorting: An alternative measure of class formation? *The Psychological Record,**65*, 615–625. 10.1007/s40732-015-0132-5

[CR7] Barnes-Holmes, D., Barnes-Holmes, Y., & Cullinan, V. (2000). Relational frame theory and Skinner’s Verbal Behavior: A possible synthesis. *The Behavior Analyst,**23*(1), 69–84. 10.1007/BF03392000 22478339 10.1007/BF03392000 PMC2731367

[CR8] Barnes-Holmes, D., & Harte, C. (2022). Relational frame theory 20 years on: The Odysseus voyage and beyond. *Journal of the Experimental Analysis of Behavior,**117*(2), 240–266. 10.1002/jeab.733 35014700 10.1002/jeab.733

[CR9] Barnes-Holmes, D., Hayes, S. C., & Dymond, S. (2001a). Self and self-directed rules. In S. C. Hayes, D. Barnes-Holmes, & B. Roche (Eds.), *Relational frame theory* (pp. 119–139). Kluwer Academic. 10.1007/0-306-47638-X_7

[CR10] Barnes-Holmes, D., O’Hora, D., Roche, B., Hayes, S. C., Bissett, R. T., & Lyddy, F. (2001b). Understanding and verbal regulation. In S. C. Hayes, D. Barnes-Holmes, & B. Roche (Eds.), *Relational frame theory* (pp. 103–117). Kluwer Academic. 10.1007/0-306-47638-X_6

[CR11] Belisle, J. (2020). Model dependent realism and the rule-governed behavior of behavior analysts: Applications to derived relational responding. *Perspectives on Behavior Science,**43*(2), 321–342. 10.1007/s40614-020-00247-x 32647785 10.1007/s40614-020-00247-x PMC7316928

[CR12] Benitez, P., & Domeniconi, C. (2016). Use of a computerized reading and writing teaching program for families of students with intellectual disabilities. *The Psychological Record,**66*(1), 127–138. 10.1007/s40732-015-0158-8

[CR13] Benitez, P., & Domeniconi, C. (2023). Equivalence-based instruction to teaching reading by families and teachers students with autism and/or intellectual disabilities. *Behavioral Interventions,**38*(3), 861–880. 10.1002/bin.1932

[CR14] Bortoloti, R., & de Rose, J. C. (2012). Equivalent stimuli are more strongly related after training with delayed matching than after simultaneous matching: A study using the Implicit Relational Assessment Procedure (IRAP). *The Psychological Record,**62*(1), 41–54. 10.1007/BF03395785

[CR15] Brodsky, J., & Fienup, D. M. (2018). Sidman goes to college: A meta-analysis of equivalence-based instruction in higher education. *Perspectives on Behavior Science,**41*(1), 95–119. 10.1007/s40614-018-0150-0 31976392 10.1007/s40614-018-0150-0 PMC6701485

[CR16] Cassidy, S., Roche, B., Colbert, D., Stewart, I., & Grey, I. M. (2016). A relational frame skills training intervention to increase general intelligence and scholastic aptitude. *Learning & Individual Differences,**47*, 222–235. 10.1016/j.lindif.2016.03.001

[CR17] Clayton, M. C., & Hayes, L. J. (1999). Conceptual differences in the analysis of stimulus equivalence. *The Psychological Record,**49*(1), 145–161. 10.1007/BF03395312

[CR18] Clayton, M. C., & Hayes, L. J. (2004). A comparison of match-to-sample and respondent-type training of equivalence classes. *The Psychological Record,**54*, 579–602. 10.1007/BF03395493

[CR19] Conceição, D. B., Greer, R. D., & Moschella, J. L. (2022). A general outline of the verbal behavior developmental theory. *Revista Brasileira de Terapia Comportamental e Cognitiva*, *24*, 1–39. 10.31505/rbtcc.v24i1.1646

[CR20] de Almeida, J. H., Cortez, M. D., & de Rose, J. C. (2020). The effects of monitoring on children’s rule-following in a computerized procedure. *Analysis of Verbal Behavior,**36*(2), 295–307. 10.1007/s40616-020-00130-5 33381384 10.1007/s40616-020-00130-5 PMC7736376

[CR21] de Rose, J. C., de Souza, D. G., Rossito, A. L., & de Rose, T. (1992). Stimulus equivalence and generalization in reading after matching to sample by exclusion. In S. C. Hayes & L. J. Hayes (Eds.), *Understanding verbal relations* (pp. 69–82). Context Press.

[CR22] de Rose, J. C., de Souza, D. G., & Hanna, E. S. (1996). Teaching reading and spelling: Exclusion and stimulus equivalence. *Journal of Applied Behavior Analysis,**29*(4), 451–469. 10.1901/jaba.1996.29-451 16795892 10.1901/jaba.1996.29-451 PMC1284016

[CR23] de Souza, D. G., de Rose, J. C., Faleiros, T. C., Bortoloti, R., Hanna, E. S., & McIlvane, W. J. (2009). Teaching generative reading via recombination of minimal textual units: A legacy of verbal behavior to children in Brazil. *International Journal of Psychology & Psychological Therapy,**9*(1), 19–44. 19960112 PMC2786216

[CR24] Debert, P., Matos, M. A., & McIlvane, W. (2007). Conditional relations with compound abstract stimuli using a go/no-go procedure. *Journal of the Experimental Analysis of Behavior,**87*(1), 89–96. 10.1901/jeab.2007.46-05 17345953 10.1901/jeab.2007.46-05 PMC1790877

[CR25] Dickins, D. W., & Bentall, R. P. (1996). Learning names may help to make the right connections. *Journal of the Experimental Analysis of Behavior,**65*(1), 259–261. 10.1901/jeab.1996.65-259 16812786 10.1901/jeab.1996.65-259 PMC1350079

[CR26] Dymond, S., May, R. J., Munnelly, A., & Hoon, A. E. (2010). Evaluating the evidence base for relational frame theory: A citation analysis. *The Behavior Analyst,**33*(1), 97–117. 10.1007/BF03392206 22479129 10.1007/BF03392206 PMC2867509

[CR27] Edwards, D. J., McEnteggart, C., Barnes-Holmes, Y., Lowe, R., Evans, N., & Vilardaga, R. (2017). The impact of mindfulness and perspective-taking on implicit associations toward the elderly: A relational frame theory account. *Mindfulness,**8*(6), 1615–1622. 10.1007/s12671-017-0734-x 29399210 10.1007/s12671-017-0734-x PMC5796557

[CR28] Fields, L. (1996). The evidence for naming as a cause or facilitator of equivalence class formation. *Journal of the Experimental Analysis of Behavior,**65*(1), 279–282. 10.1901/jeab.1996.65-279 16812791 10.1901/jeab.1996.65-279 PMC1350086

[CR29] Fields, L. (2023). Under-explored effects of structure on equivalence class formation: A commentary. *The Psychological Record,**73*(3), 507–512. 10.1007/s40732-023-00552-2

[CR30] Fienup, D. M., & Brodsky, J. (2020). Equivalence-based instruction: Designing instruction using stimulus equivalence. In M. Fryling, R. A. Rehfeldt, J. Tarbox, & L. J. Hayes (Eds.), *Applied behavior analysis of language and cognition: Core concepts and principles for practitioners* (Vol. 1, pp. 157–173). Context Press.

[CR31] Frampton, S. E., Axe, J. B., & Miguel, C. F. (2023). The effects of note taking as a visual mediation strategy on the formation of equivalence classes. *Journal of the Experimental Analysis of Behavior,**119*(3), 429–447. 10.1002/jeab.844 37000046 10.1002/jeab.844

[CR32] Galizio, M. (1979). Contingency-shaped and rule-governed behavior: Instructional control of human loss avoidance. *Journal of the Experimental Analysis of Behavior,**31*(1), 53–70. 10.1901/jeab.1979.31-53 16812123 10.1901/jeab.1979.31-53 PMC1332789

[CR33] Gilmore, A., Barnes-Holmes, D., & Sivaraman, M. (2024). A modern collaborative behavior analytic approach to incidental naming. *Perspectives on Behavior Science, 47*(3), 581–601. 10.1007/s40614-024-00399-0 10.1007/s40614-024-00399-0 PMC11411027 39309234

[CR34] Golfeto, R. M., & Postalli, L. M. M. (2021). Ensino de palavras irregulares por meio do programa de ensino aprendendo a ler e a escrever em pequenos passos. In *Contribuições da análise do comportamento para a compreensão da leitura e escrita: aspectos históricos, conceituais e procedimentos de ensino* (Vol. 1, pp. 191–224). Faculdade de Filosofia e Ciências. 10.36311/2021.978-65-5954-075-4.p191-224

[CR35] Greer, R. D., Dudek, J., & Chang, H. (2024). Observation, language learning, and development: The verbal behavior development theory. *The Psychological Record*. 10.1007/s40732-024-00585-1

[CR36] Greer, R. D., & Ross, D. E. (2008). *Verbal behavior analysis: Inducing and expanding new verbal capabilities in children with language delays* (R. D. Greer & D. E. Ross, Eds.). Pearson Education.

[CR37] Greer, R. D., & Speckman, J. (2009). The integration of speaker and listener responses: A theory of verbal development. *The Psychological Record,**59*(3), 449–488. 10.1007/BF03395674

[CR38] Gross, A. C., & Fox, E. J. (2009). Relational frame theory: An overview of the controversy. *Analysis of Verbal Behavior,**25*(1), 87–98. 10.1007/BF03393073 22477432 10.1007/BF03393073 PMC2779078

[CR39] Harte, C., Barnes-Holmes, D., Barnes-Holmes, Y., & Kissi, A. (2020). The study of rule-governed behavior and derived stimulus relations: Bridging the gap. *Perspectives on Behavior Science,**43*(2), 361–385. 10.1007/s40614-020-00256-w 32647787 10.1007/s40614-020-00256-w PMC7316874

[CR40] Hawkins, E., Gautreaux, G., & Chiesa, M. (2018). Deconstructing common bidirectional naming: A proposed classification framework. *Analysis of Verbal Behavior,**34*, 44–61. 10.1007/s40616-018-0100-7 31976214 10.1007/s40616-018-0100-7 PMC6702485

[CR41] Hayes, L. J. (1991a). Substitution and reference. In L. J. Hayes & P. N. Chase (Eds.), *Dialogues on verbal behavior: The First International Institute on Verbal Relations* (pp. 3–14). Context Press.

[CR42] Hayes, S. C. (1989). *Rule-governed behavior: Cognition, contingencies, and instructional control* (S. C. Hayes, Ed.). Plenum Press. 10.1007/978-1-4757-0447-1

[CR43] Hayes, S. C. (1991). A relational control theory of stimulus equivalence. In . In L. J. Hayes & P. N. Chase (Eds.), *Dialogues on verbal behavior* (Vol. 1, pp. ). Context Press.

[CR44] Hayes, S. C. (1996). Developing a theory of derived stimulus relations. *Journal of the Experimental Analysis of Behavior,**65*(1), 309–311. 10.1901/jeab.1996.65-309 8583202 10.1901/jeab.1996.65-309 PMC1350097

[CR45] Hayes, S. C., & Barnes, D. (1997). Analyzing derived stimulus relations requires more than the concept of stimulus class. *Journal of the Experimental Analysis of Behavior,**68*(2), 235–244. 10.1901/jeab.1997.68-235 16812857 10.1901/jeab.1997.68-235 PMC1284629

[CR46] Hayes, S. C., Barnes-Holmes, D., & Roche, B. (2001a). *Relational frame theory: A post-Skinnerian account of human language and cognition*. Plenum Press. 10.1016/s0065-2407(02)80063-5 11605362

[CR47] Hayes, S. C., Brownstein, A. J., Zettle, R. D., Rosenfarb, I., & Korn, Z. (1986). Rule-governed behavior and sensitivity to changing consequences of responding. *Journal of the Experimental Analysis of Behavior,**45*(3), 237–256. 10.1901/jeab.1986.45-237 16812448 10.1901/jeab.1986.45-237 PMC1348236

[CR48] Hayes, S. C., Gifford, E. V., Townsend, R. C., & Barnes-Holmes, D. (2001b). Thinking, problem-solving, and pragmatic verbal analysis. In S. C. Hayes, D. Barnes-Holmes, & B. Roche (Eds.), *Relational frame theory* (pp. 87–101). Springer US. 10.1007/0-306-47638-X_5

[CR49] Hayes, S. C., & Hayes, L. J. (1992). *Understanding verbal relations: The Second and Third International Institute on Verbal Relations*. Context Press.

[CR50] Hayes, S. C., & Hofmann, S. G. (2023). A biphasic relational approach to the evolution of human consciousness. *International Journal of Clinical & Health Psychology,**23*(4), 100380. 10.1016/j.ijchp.2023.100380 36937548 10.1016/j.ijchp.2023.100380 PMC10017357

[CR51] Hayes, S. C., Law, S., Assemi, K., Falletta-Cowden, N., Shamblin, M., Burleigh, K., Olla, R., Forman, M., & Smith, P. (2021). Relating is an operant: A fly over of 35 years of RFT research. *Perspectivas Em Análise Do Comportamento*. 10.18761/PAC.2021.v12.RFT.02

[CR52] Hayes, S. C., & Sanford, B. T. (2014). Cooperation came first: Evolution and human cognition. *Journal of the Experimental Analysis of Behavior,**101*(1), 112–129. 10.1002/jeab.64 24318964 10.1002/jeab.64

[CR53] Horne, P. J., & Lowe, C. F. (1996). On the origins of naming and other symbolic behavior. *Journal of the Experimental Analysis of Behavior,**65*(1), 185–241. 10.1901/jeab.1996.65-185 16812780 10.1901/jeab.1996.65-185 PMC1350072

[CR54] Horne, P. J., Lowe, C. F., & Randle, V. R. L. (2004). Naming and categorization in young children: II. Listener behavior training. *Journal of the Experimental Analysis of Behavior*, *81*(3), 267–288. 10.1901/jeab.2004.81-267 10.1901/jeab.2004.81-267 PMC1284985 15357510

[CR55] Hranchuk, K., & Greer, R. D. (2024). General reflections on interactions between cross-disciplinary theories of human language development. *The Psychological Record*. 10.1007/s40732-024-00588-y

[CR56] Keller, F. S. (1968). Good-bye, teacher …. *Journal of Applied Behavior Analysis,**1*(1), 79–89. 10.1901/jaba.1968.1-79 16795164 10.1901/jaba.1968.1-79 PMC1310979

[CR57] Lipkens, R., Hayes, S. C., & Hayes, L. J. (1993). Longitudinal study of the development of derived relations in an infant. *Journal of Experimental Child Psychology,**56*(2), 201–239. 10.1006/jecp.1993.1032 8245768 10.1006/jecp.1993.1032

[CR58] Lowe, C. F., & Horne, P. J. (1996). Reflections on naming and other symbolic behavior. *Journal of the Experimental Analysis of Behavior,**65*(1), 315–340. 10.1901/jeab.1996.65-315 16812801 10.1901/jeab.1996.65-315 PMC1350099

[CR59] Lowe, C. F., Horne, P. J., Harris, F. D. A., & Randle, V. R. L. (2002). Naming and categorization in young children: Vocal tact training. *Journal of the Experimental Analysis of Behavior,**78*(3), 527–549. 10.1901/jeab.2002.78-527 12507018 10.1901/jeab.2002.78-527 PMC1284914

[CR60] Lowe, C. F., Horne, P. J., & Hughes, J. C. (2005). Naming and categorization in young children: III. Vocal tact training and transfer of function. *Journal of the Experimental Analysis of Behavior*, *83*(1), 47–65. 10.1901/jeab.2005.31-04 10.1901/jeab.2005.31-04 PMC1193700 15762380

[CR61] Lowenkron, B. (1998). Some logical functions of joint control. *Journal of the Experimental Analysis of Behavior,**69*(3), 327–354. 10.1901/jeab.1998.69-327 9599452 10.1901/jeab.1998.69-327 PMC1284660

[CR62] Lowenkron, B. (2006). An introduction to joint control. *Analysis of Verbal Behavior,**22*, 123–127. 10.1007/BF03393034 22477351 10.1007/BF03393034 PMC2774602

[CR63] Lucchesi, F. D. M., Almeida-Verdu, A. C. M., Bolsoni-Silva, A. T., Buffa, M. J. M. B., & de Souza, D. G. (2022). Speech accuracy and reading in children with cochlear implants. *The Psychological Record,**72*(4), 697–711. 10.1007/s40732-022-00518-w

[CR64] Mackay, H. A., & Sidman, M. (1984). Teaching new behavior via equivalence relations. In B. Sperber, C. MacCauley, & P. H. Brookes (Eds.), *Learning and cognition in the mentally retarded* (pp. 493–513). Lawrence Erlbaum Associates.

[CR65] Marin, R., & Fienup, D. M. (2024). Relating in the wild: Toward an analysis of equivalence relations under more naturalistic conditions. *Perspectives on Behavior Science,**47*, 603–626. 10.1007/s40614-024-00420-6 39309240 10.1007/s40614-024-00420-6 PMC11411042

[CR66] McIlvane, W. J., & Stoddard, T. (1981). Acquisition of matching-to-sample performances in severe retardation: Learning by exclusion. *Journal of Intellectual Disability Research,**25*(1), 33–48. 10.1111/j.1365-2788.1981.tb00091.x 10.1111/j.1365-2788.1981.tb00091.x 6454002

[CR67] Melchiori, L. E., de Souza, D. G., & de Rose, J. C. (2000). Reading, equivalence, and recombination of units: A replication with students with different learning histories. *Journal of Applied Behavior Analysis,**33*(1), 97–100. 10.1901/jaba.2000.33-97 10738958 10.1901/jaba.2000.33-97 PMC1284228

[CR68] Michael, J. (1984). Verbal behavior. *Journal of the Experimental Analysis of Behavior,**42*(3), 363–376. 10.1901/jeab.1984.42-363 16812395 10.1901/jeab.1984.42-363 PMC1348108

[CR69] Michael, J. (1996). Separate repertoires or naming? *Journal of the Experimental Analysis of Behavior,**65*(1), 296. 10.1901/jeab.1996.65-296 16812798 10.1901/jeab.1996.65-296 PMC1350093

[CR70] Miguel, C. F. (2016). Common and intraverbal bidirectional naming. *Analysis of Verbal Behavior,**32*(2), 125–138. 10.1007/s40616-016-0066-2 30800621 10.1007/s40616-016-0066-2 PMC6381345

[CR71] Miguel, C. F. (2018). Problem-solving, bidirectional naming, and the development of verbal repertoires. *Behavior Analysis: Research & Practice,**18*(4), 340–353. 10.1037/bar0000110

[CR72] Murphy, C., & Barnes-Holmes, D. (2009). Establishing derived manding for specific amounts with three children: An attempt at synthesizing Skinner’s verbal behavior with relational frame theory. *The Psychological Record,**59*(1), 75–91. 10.1007/BF03395650

[CR73] Murphy, C., Lyons, K., Kelly, M., Barnes-Holmes, Y., & Barnes-Holmes, D. (2019). Using the Teacher IRAP (T-IRAP) interactive computerized programme to teach complex flexible relational responding with children with diagnosed autism spectrum disorder. *Behavior Analysis in Practice,**12*, 52–65. 10.1007/s40617-018-00302-9 30918770 10.1007/s40617-018-00302-9 PMC6411535

[CR74] O’Connor, M., Farrell, L., Munnelly, A., & McHugh, L. (2017). Citation analysis of relational frame theory: 2009–2016. *Journal of Contextual Behavioral Science,**6*(2), 152–158. 10.1016/j.jcbs.2017.04.009

[CR75] Parrott, L. J. (1984). Listening and understanding. *The*. *Behavior Analyst,**7*(1), 29–39. 10.1007/BF03391883 22478594 10.1007/BF03391883 PMC2742003

[CR76] Perez, W. F. (2023). Skinner and relational frame theory: Integrating units of analysis on a continuum of complexity. *The Psychological Record*. 10.1007/s40732-023-00559-9

[CR77] Petursdottir, A. I. (2018). The current status of the experimental analysis of verbal behavior. *Behavior Analysis: Research & Practice,**18*(2), 151–168. 10.1037/bar0000109

[CR78] Petursdottir, A. I. (2023). To dismantle or not to dismantle: Components of derived relational responding. *The Psychological Record*. 10.1007/s40732-023-00573-x

[CR79] Petursdottir, A. I., & Devine, B. (2017). The impact of verbal behavior on the scholarly literature from 2005 to 2016. *The Analysis of Verbal Behavior, 33*(2), 212–228. 10.1007/s40616-017-0089-3 10.1007/s40616-017-0089-3 PMC6381327 30854298

[CR80] Pilgrim, C. (1996). Can the naming hypothesis be falsified? *Journal of the Experimental Analysis of Behavior,**65*(1), 284–286. 10.1901/jeab.1996.65-284 16812793 10.1901/jeab.1996.65-284 PMC1350088

[CR81] Pilgrim, C. (2020). Equivalence-based instruction. In J. O. Cooper, T. E. Heron, & W. L. Heward (Eds.), *Applied behavior analysis* (3rd ed., pp. 442–496). Pearson.

[CR82] Pomorska, K., Ostaszewski, P., Barnes-Holmes, Y., & McEnteggart, C. (2021). Protocols for assessing derived relations in typically developing children and children with autism spectrum disorder. *The Psychological Record,**71*(3), 435–460. 10.1007/s40732-020-00442-x

[CR83] Regaço, A., Zapparoli, H. R., Aggio, N. M., Silveira, M. V., & Arntzen, E. (2023). Maintenance of stimulus equivalence classes: A bibliographic review. *The Psychological Record,**73*, 1–11. 10.1007/s40732-023-00535-3

[CR84] Ribeiro, G. W., Kawasaki, H. N., Menzori, L. R., Amd, M., de Rose, J. C., & de Souza, D. G. (2020). Emergent reading via stimulus pairing with orientation response. *The Psychological Record,**70*(3), 397–410. 10.1007/s40732-020-00398-y

[CR85] Rosales-Ruiz, J., & Baer, D. M. (1997). Behavioral cusps: A developmental and pragmatic concept for behavior analysis. *Journal of Applied Behavior Analysis,**30*(3), 533–544. 10.1901/jaba.1997.30-533 9316263 10.1901/jaba.1997.30-533 PMC1284066

[CR86] Saunders, K. J., & Spradlin, J. E. (1996). Naming and equivalence relations. *Journal of the Experimental Analysis of Behavior,**65*(1), 304–308. 10.1901/jeab.1996.65-304 8583201 10.1901/jeab.1996.65-304 PMC1350096

[CR87] Schlinger, H. D., Jr., & Blakely, E. (2024). A mediational theory of equivalence relations and transformation of function. *Journal of the Experimental Analysis of Behavior,**122*(2), 207–223. 10.1002/jeab.4204 39118281 10.1002/jeab.4204

[CR88] Sidman, M. (1971). Reading and auditory-visual equivalence. *Journal of Speech & Hearing Research,**14*(1), 5–13. 5550631 10.1044/jshr.1401.05

[CR89] Sidman, M. (1994). *Equivalence relations and behavior: A research story.* Authors Cooperative.

[CR90] Sidman, M. (2000). Equivalence relations and the reinforcement contingency. *Journal of the Experimental Analysis of Behavior,**74*(1), 127–146. 10.1901/jeab.2000.74-127 10966100 10.1901/jeab.2000.74-127 PMC1284788

[CR91] Sidman, M., & Tailby, W. (1982). Conditional discrimination vs. matching to sample: An expansion of the testing paradigm. *Journal of the Experimental Analysis of Behavior*, *37*(1), 5–22. 10.1901/jeab.1982.37-5 10.1901/jeab.1982.37-5 PMC1333115 7057129

[CR92] Sidman, M., Willson-Morris, M., & Kirk, B. (1986). Matching-to-sample procedures and the development of equivalence relations: The role of naming. *Analysis & Intervention in Developmental Disabilities,**6*(1–2), 1–19. 10.1016/0270-4684(86)90003-0

[CR93] Sivaraman, M., & Barnes-Holmes, D. (2023). Naming: What do we know so far? A systematic review. *Perspectives on Behavior Science,**46*(3–4), 585–615. 10.1007/s40614-023-00374-1 38144546 10.1007/s40614-023-00374-1 PMC10733260

[CR94] Sivaraman, M., Barnes-Holmes, D., Greer, R. D., Fienup, D. M., & Roeyers, H. (2023). Verbal behavior development theory and relational frame theory: Reflecting on similarities and differences. *Journal of the Experimental Analysis of Behavior,**119*(3), 539–553. 10.1002/jeab.836 36808741 10.1002/jeab.836

[CR95] Sivaraman, M., Barnes-Holmes, D., & Roeyers, H. (2021). Nonsimultaneous stimulus presentations and their role in listener naming. *Journal of the Experimental Analysis of Behavior,**116*(3), 300–313. 10.1002/jeab.715 34542178 10.1002/jeab.715

[CR96] Skinner, B. F. (1957). *Verbal behavior*. Appleton-Century-Crofts.

[CR97] Skinner, B. F. (1966). An operant analysis of problem solving. In B. Kleinmuntz (Ed.), *Problem-solving: Research, method, and theory* (pp. 225–257). Wiley.

[CR98] Skinner, B. F. (1969). *Contingencies of reinforcement: A theoretical analysis*. BF Skinner Foundation.

[CR99] Stewart, I., & Barnes-Holmes, D. (2004). Relational frame theory and analogical reasoning: Empirical investigations. *International Journal of Psychology & Psychological Therapy,**4*(2), 241–262.

[CR100] Sundberg, C. T., Sundberg, M. L., & Michael, J. (2018). Covert verbal mediation in arbitrary matching to sample. *Journal of the Experimental Analysis of Behavior,**109*(3), 600–623. 10.1002/jeab.434 29733443 10.1002/jeab.434

[CR101] Sundberg, M. L., & Michael, J. (2001). The benefits of Skinner’s analysis of verbal behavior for children with autism. *Behavior Modification,**25*(5), 698–724. 10.1177/0145445501255003 11573336 10.1177/0145445501255003

[CR102] Zapparoli, H. R., Marin, R., & Harte, C. (2021). Rule-governed behavior: An ongoing RFT-based operant analysis. *Perspectivas Em Análise Do Comportamento, 12*(1), 197–213. 10.18761/PAC.2021.v12.RFT.09

